# C-reactive protein and association with disease severity in hospitalized adult patients with Lassa fever in Nigeria

**DOI:** 10.1016/j.ijregi.2024.100506

**Published:** 2024-12-05

**Authors:** Ebenezer Oseremen Dic-Ijiewere, Danny Asogun, Festus Oloruntoba Okojie, Adoghe Patricia Omono, Okpunu Eseoleleti Christopher, Adam Zumla, Rizwan Ahmed, Faith Huemomen Unuabonah, Joseph Okoeguale, Cyril Erameh, Ephraim Ogbainin, Sylvanus Okogbenin, Reuben Eifediyi, Linzy Elton, Isobella Honeyborne, John Tembo, Francine Ntoumi, Najmul Haider, Timothy D. McHugh, Alimuddin Zumla

**Affiliations:** 1 Department of Chemical Pathology, College of Medicine, Ambrose Alli University, Ekpoma, Nigeria; 2 Institute of Viral and Emergent Pathogens, Irrua Specialist Teaching Hospital, Ekpoma, Nigeria; 3 Department of Community medicine, Irrua Specialist Teaching Hospital, Irrua, and Faculty of Clinical Sciences, Ambrose Alli University, Ekpoma, Nigeria; 4 Department of Haematology, Irrua Specialist Teaching Hospital, Irrua & Department of Haematology, Ambrose Alli University, Ekpoma, Nigeria; 5 Royal Bolton Hospital, Bolton NHS Foundation Trust, Bolton, United Kingdom; 6 Department of Haematology and Transfusion Science, Ambrose Alli University, Ekpoma, Nigeria; 7 Division of Infection and Immunity, Centre for Clinical Microbiology, University College London, London, United Kingdom; 8 HerpeZ and PANDORA-D-NET, University Teaching Hospital, Lusaka, Zambia; 9 Fondation Congolaise Pour la Recherche Médicale, Brazzaville, Republic of Congo; 10 Institute for Tropical Medicine, University of Tübingen, Tübingen, Germany; 11 School of Life Sciences, Faculty of Natural Sciences, Keele University, Keele, United Kingdom; 12 National Institutes of Healthcare Research (NIHR), Biomedical Research Centre, UCL Hospitals NHS Foundation Trust, London, United Kingdom

**Keywords:** Lassa, Fever, Lassa fever virus, Biomarkers, C-reactive protein (CRP) inflammation, Disease severity

## Abstract

**Objectives:**

Lassa fever (LF) poses a significant health burden in West Africa. The pathophysiology of LF and determinants of clinical spectrum of disease remain poorly understood. We performed a study to understand the correlation of blood inflammatory marker C-reactive protein (CRP), with LF disease severity.

**Methods:**

A cross-sectional controlled study of adult patients with LF presenting to the Institute of Lassa Fever Research and Control at Irrua Specialist Teaching Hospital, Nigeria between January and April 2023. LF was confirmed using real-time polymerase chain reaction. Disease severity was classified as: severe disease = blood urea level >100 mg/dl and moderate disease = <100 mg/dl. CRP was measured using Tecan Infinite F50 ELISA system. Disease severity was correlated with CRP levels.

**Results:**

A total of 64 adult patients with LF and 60 healthy controls were enrolled. There was no difference in the mean age (37.6 vs 35.2 years, *P* = 0.10) and gender (male 53% vs 56%, *P* = 0.82). CRP levels were significantly elevated in patients with LF (mean: 36.23 mg/l, SD: 4.56, %coefficient of variation [CV]: 12.59) compared with controls (mean: 5.42 mg/l, SD: 0.53, %CV: 9.78) (*P* = 0.000). CRP levels varied with disease severity, being significantly higher in patients with severe disease (28.57 mg/l, SD = 2.34; %CV = 8.19) than in those with moderately severe disease (12.34 mg/l, SD = 0.98; %CV = 7.94); (*P* = 0.001).

**Conclusions:**

Inpatients with LF showed significant inflammation and elevated CRP levels, which correlated with disease severity. CRP could serve as a potential baseline marker in outbreak situations to trigger management decision-making to treat or not to treat considering limited ribavirin supplies. Given the inflammatory changes and correlation of elevated CRP levels with LF disease severity, larger controlled studies during outbreaks are required to assess use of CRP for more accurate triaging, including commencement of treatment.

## Highlights

• Lassa fever (LF) is a World Health Organization priority infectious disease endemic in West Africa.
• Biomarkers of LF severity are required to triage patients during outbreaks.
• Significantly elevated C-reactive protein (CRP) levels occur in patients with LF.
• CRP levels correlate with LF disease severity.
• CRP may serve as a potential baseline marker to trigger management decisions.

## Introduction

Lassa fever (LF), caused by the Lassa virus [1,2], is a lethal World Health Organization priority infectious disease for research and development [3]. Patients with Lassa virus infection present with a spectrum of clinical manifestations, from mild flu-like symptoms to severe disease, including renal failure, encephalitis, and hemorrhage, with death in up to 20% of cases [[1], [2], [3]]. Endemic to West Africa, Nigeria has reported 971 confirmed cases with 166 deaths (case fatality rate 17%) from January to October 2024, including over 30 cases among health care workers [4]. In LF-endemic west African countries where LF specific diagnostics tests and antiviral treatments are in short supply, screening and treating everyone with symptoms is unpractical. During large LF outbreaks, there is a great need for a biomarker of disease severity, which can help clinical management decision-making so that the scarce antiviral ribavirin and diagnostic test stocks are used judiciously for those in greatest need.

C-reactive protein (CRP) is a widely used clinically as a non-specific biomarker of inflammation and guide for assessing disease severity and progression [[2], [3], [4], [5]]. It is useful for clinical evaluation and management of patients with infectious and inflammatory diseases [[6], [7], [8], [9]]. CRP was extensively used during the COVID-19 pandemic in pathogenesis and prognostic studies and for clinical decision-making [7]. However, due to the dangerous nature of the Lassa virus, its high transmissibility requires strict implementation of infection control procedures and safe laboratory environment for handling blood samples. Thus, basic science and translational clinical research on LF has been hampered, and there have been scanty clinical studies using blood samples during outbreaks in humans. With enhanced laboratory capabilities at our institution, the Institute of Lassa Fever Research and Control at Irrua Specialist Teaching Hospital, we conducted a prospective cross-sectional study to assess CRP levels across the clinical spectrum of LF in southern Nigeria.

## Methods

### Study design and setting

A prospective cross-sectional controlled study conducted at the Institute of Lassa Fever Research and Control at Irrua Specialist Teaching Hospital, a leading center for LF research and care in Edo State, Nigeria. The study enrolled 64 inpatients with polymerase chain reaction (PCR)–confirmed LF and 60 matched healthy controls between December 2022 and April 2023.

### Sample collection and classification of disease severity

Plasma samples were obtained from patients who tested positive for Lassa virus using the RealStar Lassa Virus Real-Time PCR Kit 1.0 (Altona Diagnostics, Hamburg, Germany). Disease severity was categorized based on blood urea levels, with concentrations above 100 mg/dL indicating severe cases and levels below 100 mg/dl indicating moderately severe cases.

### Laboratory analysis

CRP levels were measured using the Tecan Infinite F50 ELISA microplate reader.

### Statistical analysis

Data analysis was conducted using SPSS version 21.0. The significance of differences between groups was assessed using one-way analysis of variance, chi-square test, and independent Student's *t*-tests.

## Results

### Patient demographics

The mean age of patients with LF was 37.6 years (±7.9), compared with 35.2 years (±7.9) in the control group (*P* = 0.10). Males comprised 56% of the LF group and 54% of the control group (*P* = 0.82).

### Blood urea levels

Blood urea levels used to classify disease severity were significantly elevated in the LF group, with a mean of 56.21 mg/dl (SD = 6.09; %coefficient of variation [CV] = 10.83) compared with 8.05 mg/dl (SD = 0.18; %CV = 2.24) in the control group (*P* <0.001). Urea levels were significantly elevated in severe LF cases (163.0 mg/dl, SD = 22.48; %CV = 13.79) and moderately severe cases (65.84 mg/dl, SD = 5.64; %CV = 8.57) compared with controls (*P* = 0.0001 for both comparisons).

### CRP levels

CRP levels were significantly elevated in all patients with LF, with a mean of 36.23 mg/l (SD = 4.56; %CV = 12.59), compared with 5.42 mg/l (SD = 0.53; %CV = 9.78) in healthy controls (*P* <0.001).

### Disease severity and association with CRP levels

CRP levels varied with disease severity, being significantly higher in patients with severe disease (28.57 mg/l, SD = 2.34; %CV = 8.19) than in those with moderately severe cases (12.34 mg/L, SD = 0.98; %CV = 7.94); (*P* = 0.001).

### Correlation analysis

**Figure 1:**
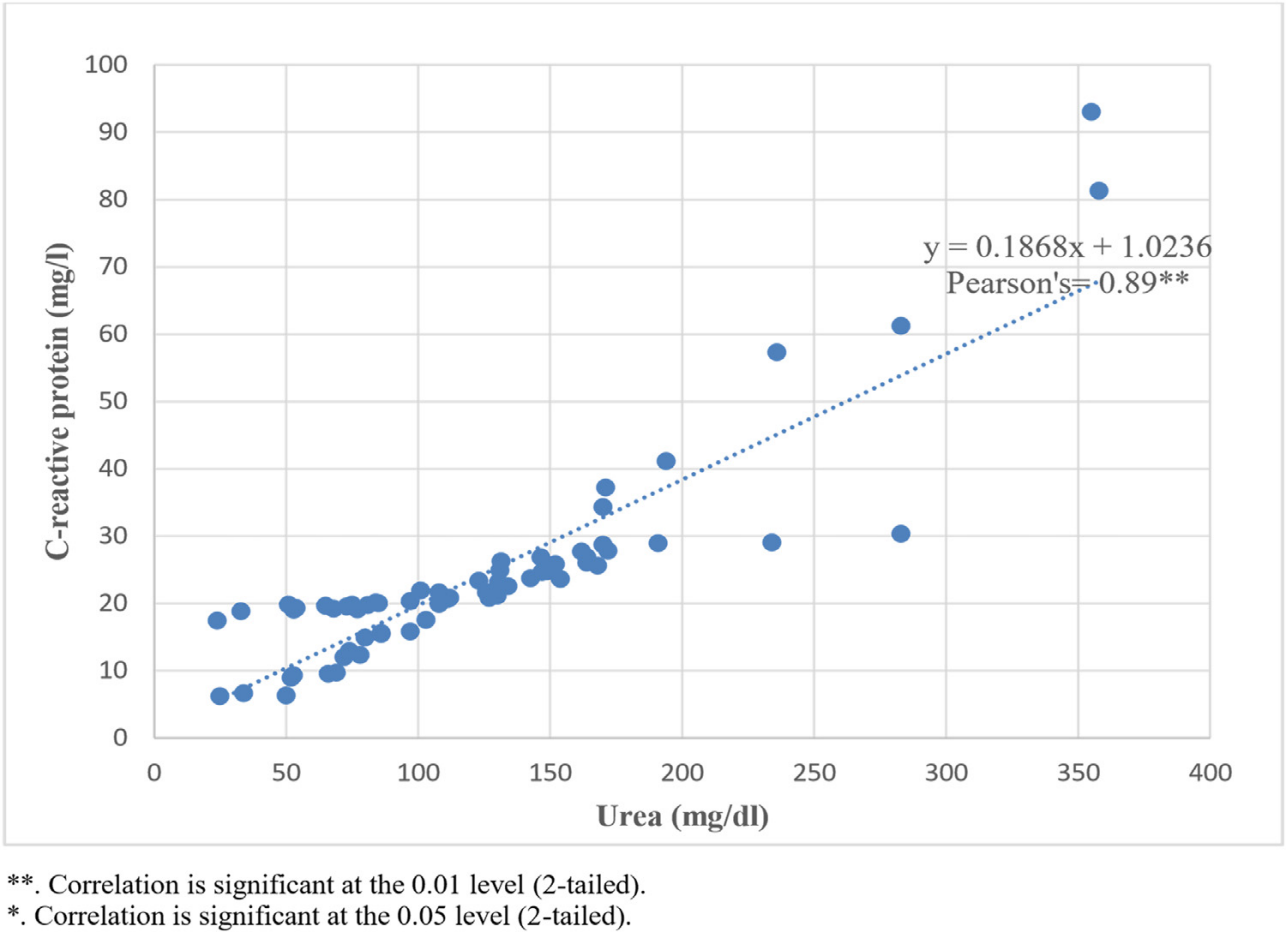
C-reactive protein levels and diseases severity. Scattered plot showing Pearson correlation between C-reactive protein and plasma urea levels. Lassa fever samples. From the results obtained, there was a strong positive correlation between C-reactive protein and plasma urea levels in Lassa fever infection (Pearson correlation = 0.89**; *P* = 0.000).

Figure 1 illustrates the correlation between plasma CRP levels and plasma urea levels in inpatients with LF, highlighting a significant positive relationship between these markers, suggesting a link between inflammation and kidney function impairment in severe cases.

## Discussion

This study, to the best of our knowledge, is the first case-controlled analysis of CRP levels in hospitalized patients with laboratory confirmed LF. We demonstrate a significant correlation of CRP levels with disease severity. Our findings show that elevated CRP is a consistent feature across the whole clinical spectrum of LF disease, with CRP levels increasing as disease severity worsens. This finding supports the hypothesis that the intensity of the inflammatory response corresponds with disease progression and outcomes [5].

During LF outbreaks, many individuals infected with Lassa virus may be asymptomatic or present with non-specific symptoms, complicating the identification of severe cases during triage [[1], [2], [3]]. Not all these individuals are tested, and, among those with fever, not all cases are due to LF. In such scenarios, a blood test showing elevated CRP levels could help identify patients who require priority attention. CRP is one of the oldest clinical biomarkers used widely in routine clinical practice worldwide [[5], [6], [7], [8], [9]], and its use increased during the COVID-19 pandemic as a marker of disease severity and prognosis [6,7]. Measuring elevated CRP levels could help prioritize those who need further diagnostic testing or urgent care, especially in settings where confirmatory diagnostics and antiviral treatments are limited. By guiding the allocation of limited diagnostic and therapeutic resources, CRP testing, which is readily available in most clinical care settings, could improve triage and management during outbreaks of viral infections [[7], [8], [9],[10], [11]]. Although CRP is a non-specific marker of inflammation and cannot pinpoint the cause of infection, it could be a useful first-line tool for triaging patients. It should be used alongside specific diagnostic tests, such as Lassa virus PCR, for a more accurate diagnosis and prognosis.

Comparatively, earlier studies in non-human primates infected with Ebola virus (EBOV), another highly pathogenic virus endemic to West Africa, showed that CRP combined with liver markers, such as Aspartate transaminase and Lactose dehydrogenase, outperformed quantitative reverse transcription polymerase chain reaction for detecting EBOV infection [10]. Although the pathophysiological mechanisms of LF and Ebola virus disease differ, these findings underscore the potential of CRP as a valuable indicator of disease severity in viral hemorrhagic fevers. Currently, the standardized criteria for initiating treatment in LF based on clinical markers or specific biomarkers are lacking [11].

To strengthen the evidence for the use of CRP in managing patients with suspected LF during large outbreaks, future research should focus on large, multicenter studies that include diverse patient populations across different regions affected by LF. Moreover, there is a need for randomized controlled trials to evaluate the use of CRP levels as a trigger for initiating specific antiviral therapies or adjunctive use of anti-inflammatory agents such as dexamethasone or other host-directed therapies such as micronutrients [3,11]. These trials would help determine whether CRP-guided treatment protocols could improve clinical outcomes, such as reduced mortality or shorter hospitalization durations. In addition, implementation studies are needed to assess the feasibility and cost-effectiveness of integrating CRP testing into triage procedures during outbreaks, particularly, in resource-limited settings. Such studies would evaluate the impact of CRP testing on reducing delays in treatment and optimizing the use of expensive diagnostic tests and antiviral drugs. These research efforts could ultimately support the development of evidence-based guidelines for using CRP alone or in combination with other markers for triaging and management of LF, thereby improving patient care and resource allocation during future outbreaks. When optimizing the allocation of limited diagnostic and therapeutic resources, CRP testing could play a significant role in improving patient care and management during future LF outbreaks.

## Conclusion

Our study showed that the blood biomarker CRP significantly correlated with LF disease severity. During large LF outbreaks, currently, there are no standardized clinical guide for the initiation of widespread screening and triage procedures for isolation and infection control, and decisions whether to utilize the scarce resources of specific Lassa virus diagnostic tests and antiviral therapies. CRP levels in patients suspected of having Lassa virus infection may serve as a potential baseline marker to guide management decision-making whether to treat or not to treat. Given the inflammatory changes and correlation of elevated CRP levels with LF disease severity, larger controlled studies during outbreaks are required to assess for more accurate triaging, including commencement of treatment.

## Funding

This study received funding from the EU-EDCTP2–EU Horizon 2020 Framework Programme, PANDORA-ID-NET grant. All authors have an academic interest in infectious diseases with pandemic potential.

## Ethics approval and consent to participate

Ethical approval was obtained from the research ethics committee of Irrua Specialist Teaching Hospital, Irrua, Nigeria.

## Author contributions

Each author made substantial contributions to PANDORA-ID-NET and its studies on viral pathogens in West Africa, including the study concept, design, conduct, data analysis, interpretation, development and review of the figure, and writing of several drafts of the manuscript. All authors have seen the final draft and approved submission. They have agreed to be personally accountable for the authors’ own contributions and ensure that questions related to the accuracy or integrity of any part of the work, even ones in which the authors were not personally involved, are appropriately investigated, resolved, and the resolution documented in the literature.

## Data availability

The data sets used and/or analyzed during the current study are available from the corresponding author on reasonable request.

## Statement

During the preparation of this work, the authors did not use AI or other writing tools.
